# Supplementary material for: Computational elements based on coupled VO2 oscillators via tunable thermal triggering
Source: Nat Commun. 2024 Jul 11;15:5820. doi: 10.1038/s41467-024-49925-3 (PMC11236964; doi:10.1038/s41467-024-49925-3)
Supplement: Supplementary file 1 — Supplementary Information [file 41467_2024_49925_MOESM1_ESM.pdf]

Supplementary information

Computational elements based on coupled VO_2 oscillators via tunable thermal triggering

Guanmin Li[1], Zhong Wang[1], Yuliang Chen[1], Jae-Chun Jeon[1]* and Stuart S. P. Parkin[1]*
[1]Max Planck Institute of Microstructure Physics, Weinberg 2, 06120 Halle (Saale), Germany
*Corresponding authors: jae-chun.jeon@mpi-halle.mpg.de, stuart.parkin@mpi-halle.mpg.de

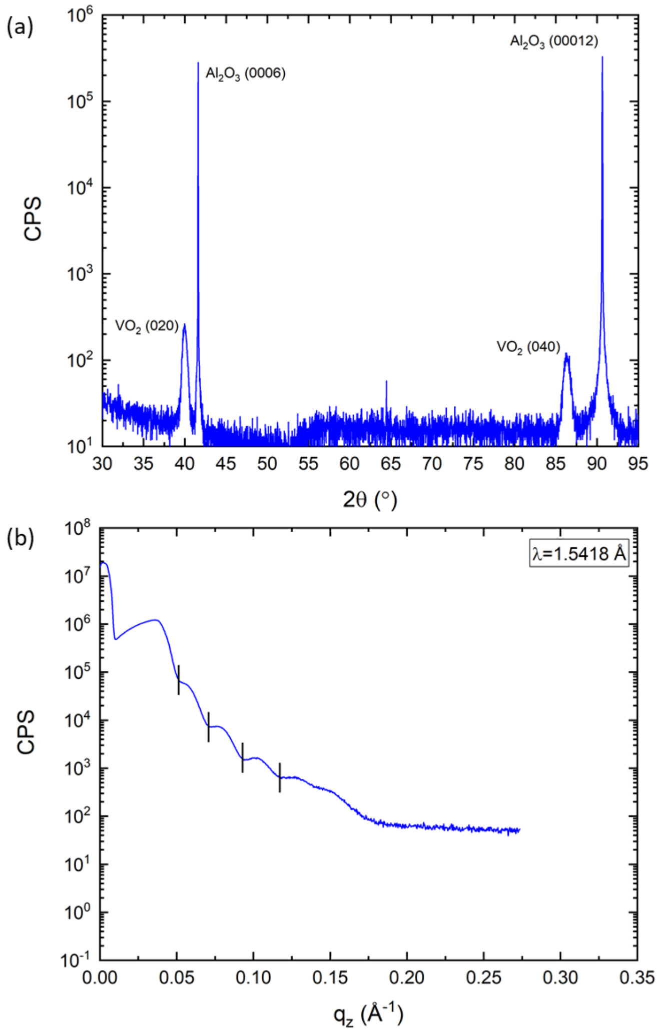

Fig. S1 | X-ray diffraction (XRD) and X-ray reflectometry (XRR) measurements of a typical VO_2 film. **a**, Theta (θ) -2Theta (2θ) scan shows (020) peak at 2θ = 39.99° and (040) peak at 2θ = 86.30°. **b**, The film thickness determined from the XRR measurement is 28.78 ± 2.54 nm.

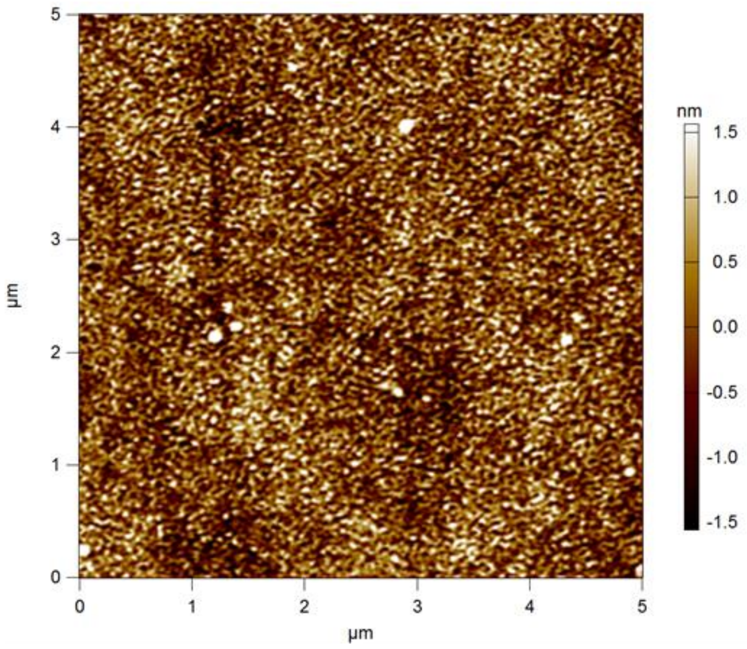

Fig. S2 | AFM scan of a typical VO_2 thin film. The RMS roughness is ~0.78 nm averaged over an area of 5 × 5 µm^2.

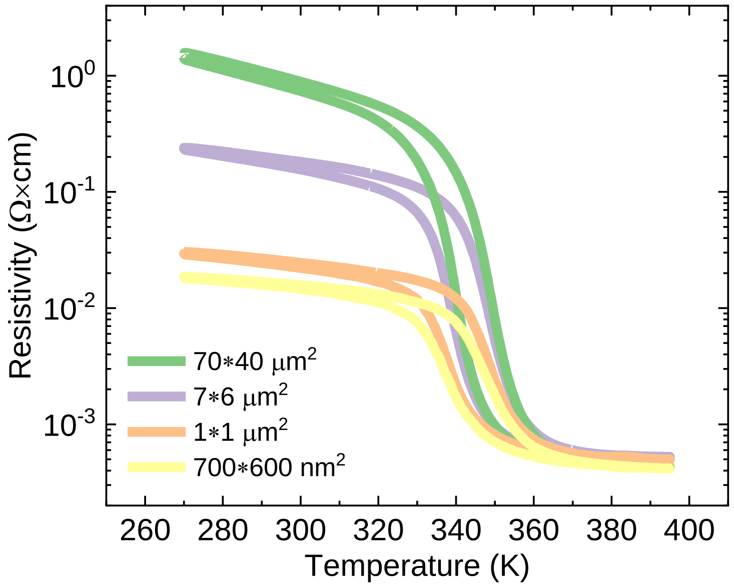

Fig. S3 | ρ-T curves of VO_2 devices with different sizes. Green: 70 × 40 µm^2, purple: 7 × 6 µm^2, orange: 1 × 1 µm^2 and yellow: 600 × 700 nm^2.

Finite element simulations of the thermal coupling between VO_2 devices

To study the steady effect of Joule heating, we numerically simulated the temperature distribution of a set of VO_2 devices each with dimensions of *w* x *w* x *t*. Here only t = 30 nm is considered. The VO_2 devices were placed on a sapphire (Al_2O_3) substrate that had the dimensions of 50 μm (length) × 50 μm (width) × 30 μm (thickness). The bottom and edges of the substrate were fixed at a constant temperature of 295 K. Only heat transfer between the sapphire and the VO_2 devices is considered. The steady-state heat transfer was modelled with:

$$-\nabla \cdot (\kappa \nabla T) = \mathrm{Q}$$

where κ, T, Q are the thermal conductivity, temperature, and heat density, respectively. The liner relationship between the power consumption of the entire device P and device width w can be solved from the above equation as:

$$P \propto 4\pi \cdot \Delta T \cdot \kappa \cdot \mathrm{w}$$

The time-dependent heat transfer was modelled with:

$$\rho c \frac{\partial T}{\partial t} - \nabla \cdot (\kappa \nabla T) = \mathrm{Q}$$

where t, ρ and c are the time, density, and heat capacity, respectively. The material parameters for Al_2O_3 [R1 - R3] and VO_2 [R4, R5] were taken from the literature in the COMSOL library.

Material	Density (g/cm^3) at 300 K	Iso pressure specific heat ($J*K^{-1}*kg^{-1}$) at 300 K	Thermal conductivity ($W*K^{-1}*m^{-1}$) at 300 K
Al_2O_3	3.989	779	34.5
VO_2	4.571	623	4.2

Supplementary data table. T1 | Material parameters for COMSOL simulations.

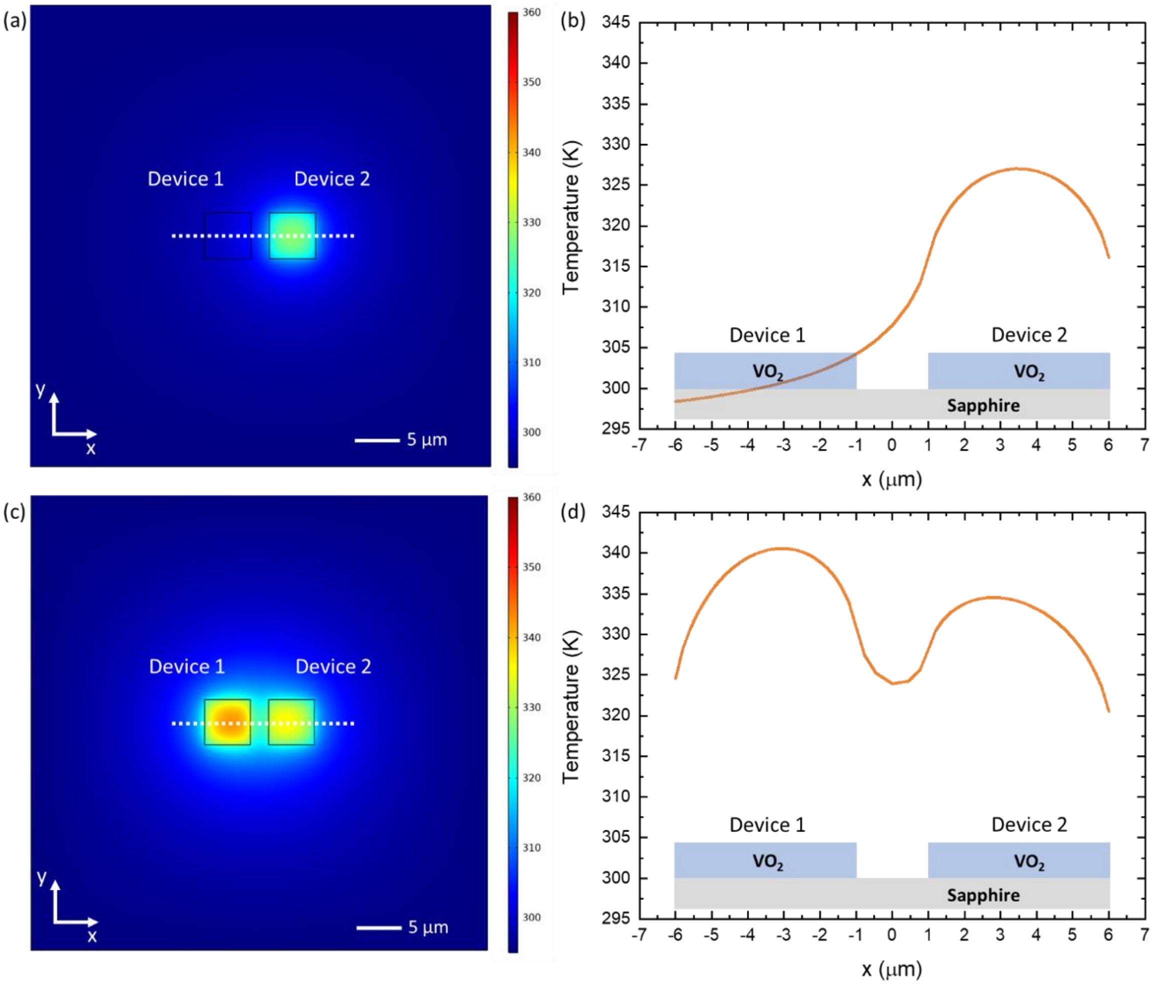

Fig. S4 | Simulation of one VO_2 device in its sub-threshold transition state that is triggered to oscillate by a neighboring oscillating VO_2 device at a 2 μm spacing. Temperature distribution of two VO_2 devices. **a**, Device 1 (5 μm × 5 μm × 30 nm) is supplied with 0 mA current (no oscillation), while device 2 (5 μm × 5 μm × 30 nm) is supplied with 2.3 mA current (sub-threshold transition state) at 295 K. Black squares correspond to the VO_2 devices. **b**, Temperature distribution versus x-axis (dashed line) in case **a**. When device 1 is turned off, the temperature of device 2 is ~327 K that is lower than the phase oscillation temperature range (330 - 360 K). **c**, Device 1 is supplied with 2.8 mA current (stable oscillation state), while device 2 is supplied with 2.3 mA current (sub-threshold transition state) at 295 K. **d**, Temperature distribution versus x-axis (dash line) in case **c**. Simulation shows that, due to the heat dissipated from device 1, the temperature of device 2 (334 K) has been raised to the phase oscillation temperature range (330 - 360 K).

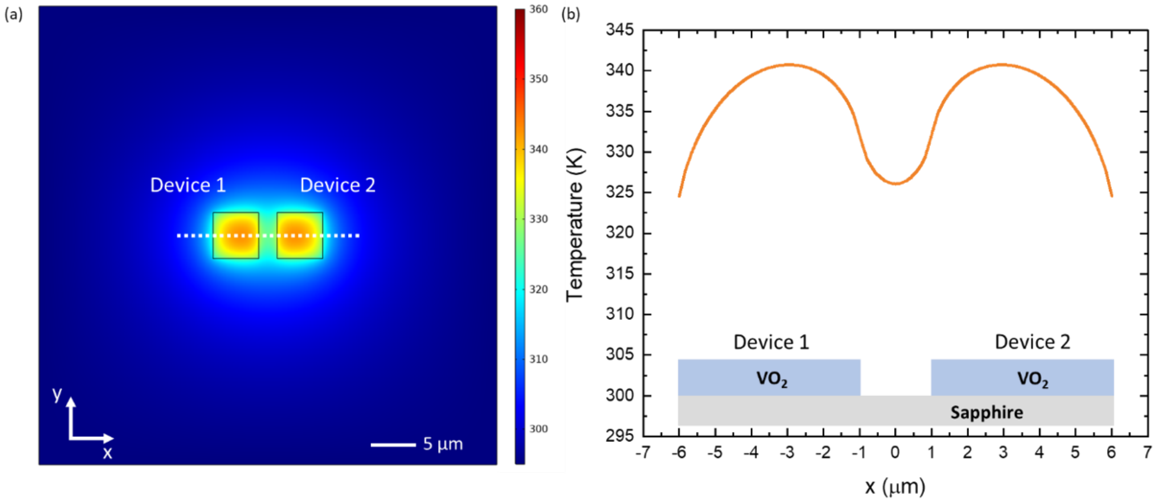

Fig. S5 | Simulation of two VO_2 devices in a stable oscillation state. a, Temperature distribution of two VO_2 devices (both 5 μm × 5 μm × 30 nm, with 2 μm spacing) when they are each supplied with 2.8 mA current ($I_1 = I_2$) at 295 K. Black squares correspond to the VO_2 devices. **b,** Temperature distribution versus x-axis (dash line). When both devices are in a stable oscillation state, their temperature (340.5 K) stays within the phase oscillation temperature range (330 - 360 K).

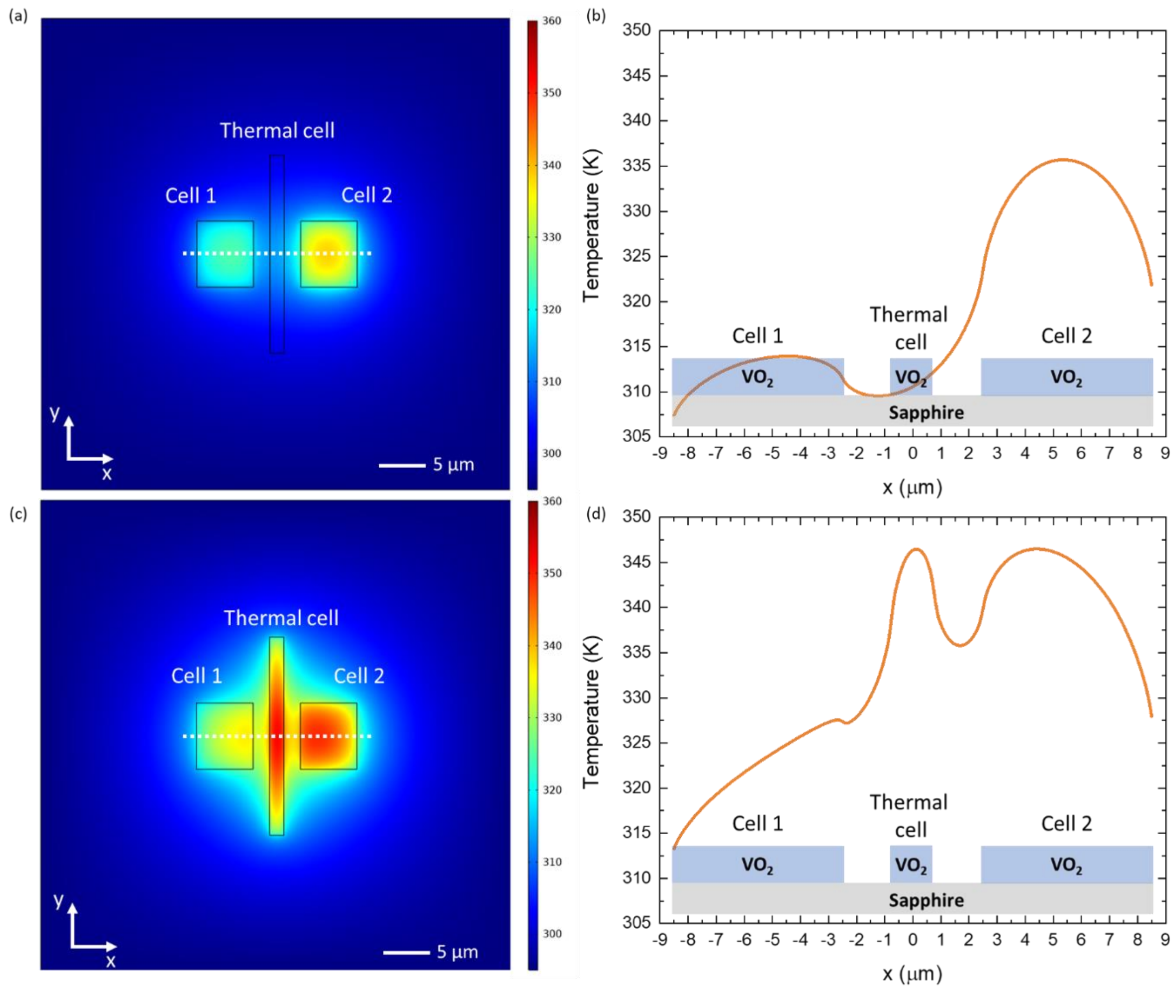

Fig. S6 | Simulation of VO_2 neuron firing mode 3. Temperature distribution of two VO_2 devices (with 5 µm spacing) and thermal cell. **a,** Device 1 (7 µm × 6 µm × 30 nm) is supplied with 1.7 mA current (no oscillation), while device 2 (7 µm × 6 µm × 30 nm) is supplied with 4 mA current (stable oscillation state) at 295 K. The thermal cell (21 µm × 1.5 µm × 30 nm) is off (I_{cell} = 0). Black squares correspond to the VO_2 devices and thermal cell. **b,** Temperature distribution versus x-axis (dash line) in case **a**. When the thermal cell is off, the temperature of device 1 (around 314 K) is lower than the phase oscillation range (330 - 360 K). **c,** Device 1 is supplied with 1.7 mA current, while device 2 is supplied with 2.3 mA current at 295 K. The thermal cell is on (I_{cell} = 2.3 mA). **d,** Temperature distribution versus x-axis (dash line) in case **c**. Simulation shows due to the heat dissipated from thermal cell, the temperature of device 2 (336 K) has been raised to the phase oscillation temperature range (330 - 360 K).

The simulation results from Fig. S6 are consistent with the experimental results in Fig. S26. When the thermal cell is turned off, we can only observe stable oscillation waveforms from

VO_2 cell 2, as shown in Fig. S21b. The weak oscillation from VO_2 cell 1 comes from the periodic heat perturbation from oscillating VO_2 cell 2 that induces the resistance of VO_2 cell 1 to slightly change. When the thermal cell is activated with I_{cell} = 2.3 mA, oscillations from both VO_2 cell 1 and cell 2 can be observed, as shown in Fig. S21a. The oscillation of the thermal cell strongly links the oscillation of cell 1 and cell 2 to its own period.

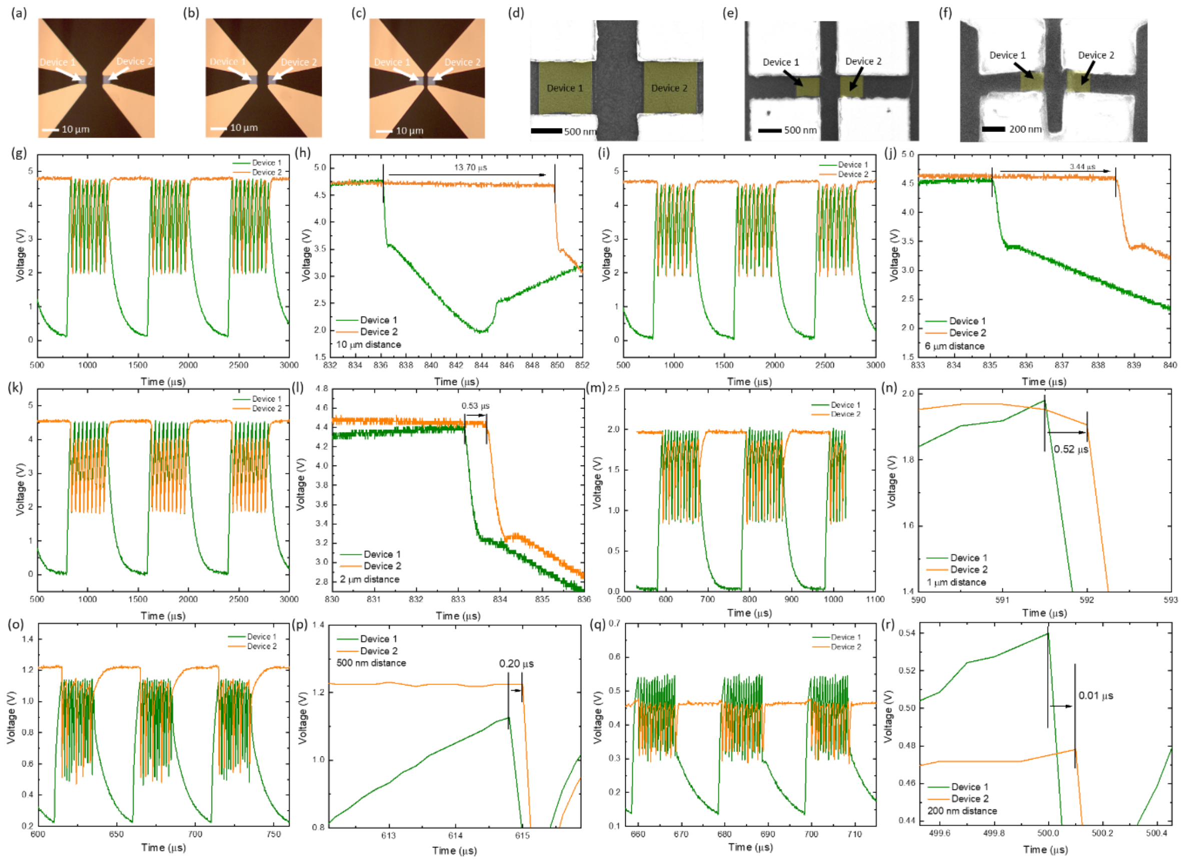

Fig. S7 | Trigger delay time for two VO_2 devices with various device spacings. Optical images of device 1 (5 × 5 µm^2) and device 2 (5 × 5 µm^2) separated by **a,** 2 µm, **b,** 6 µm, and **c,** 10 µm. SEM images of fabricated nanoscopic devices. **d**, 1 × 1 µm^2 (spacing = 1 µm), **e**, 500 × 500 nm^2 (spacing = 500 nm) and **f**, 200 × 200 nm^2 (spacing = 200 nm). Yellow shading indicates the VO_2 cells (Au contacts are in white). **g** and **h** show measurements (I_1 = 2.3 mA, I_2 = 2.8 mA) of the trigger delay time at 10 µm spacing. **i** and **j** show measurement (I_1 = 2.3 mA, I_2 = 2.8 mA) of the trigger delay time at 6 µm spacing (2 different measurements). **k** and **l** show measurements (I_1 = 2.3 mA, I_2 = 2.8 mA) of the trigger delay time at 2 µm spacing. **m** and **n** show measurements (I_1 = 420 µA, I_2 = 600 µA) of the trigger delay time at 1 µm spacing. **o** and **p** show measurements (I_1 = 460 µA, I_2 = 600 µA) of the trigger delay time at 500 nm

spacing. **q** and **r** show measurements (I_1 = 460 µA, I_2 = 600 µA) of the trigger delay time at 200 nm spacing.

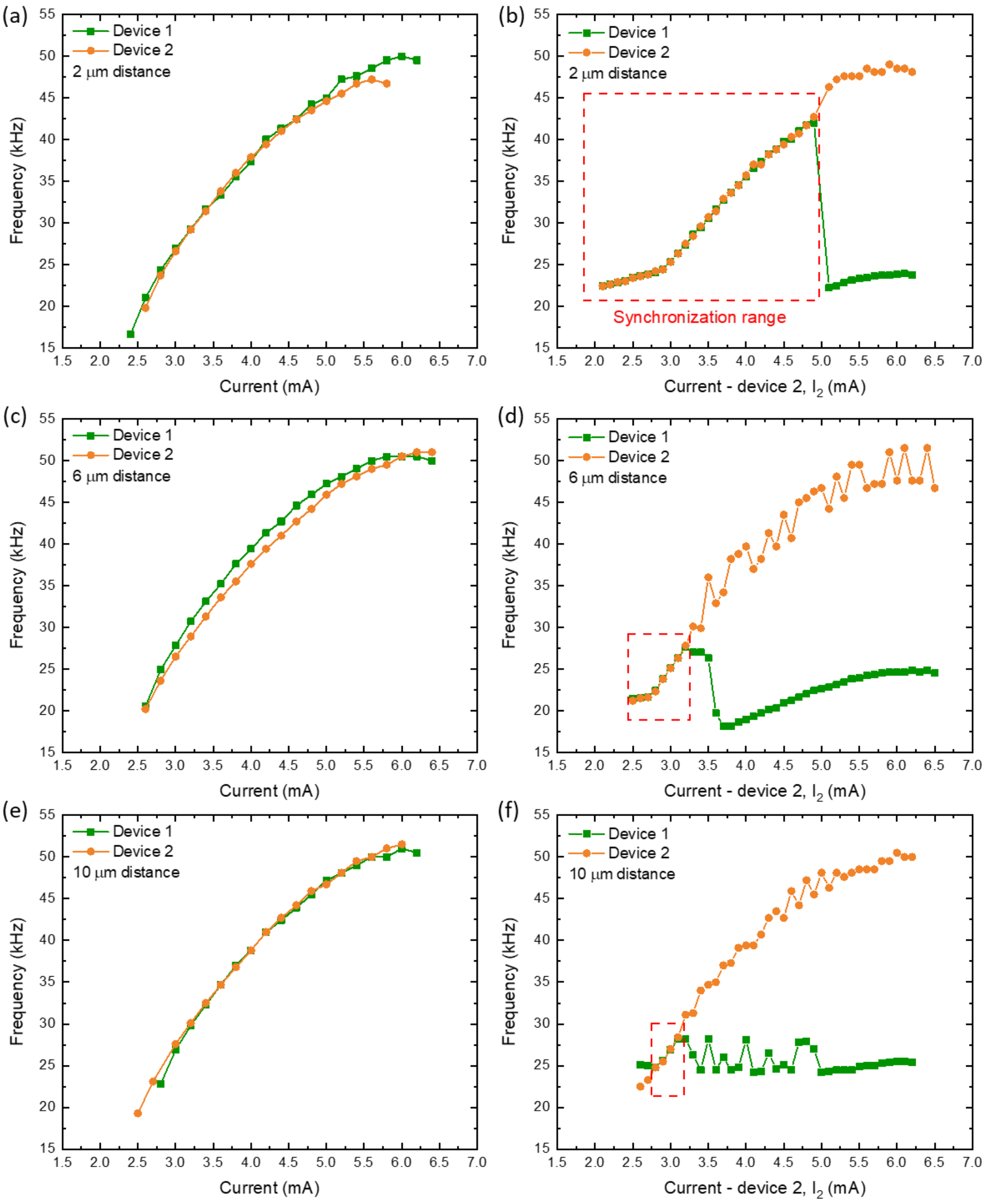

Fig. S8 | Thermal coupling strength versus device spacing. Frequency vs. applied current for device 1 (5 × 5 µm^2) and device 2 (5 × 5 µm^2) when they are oscillating independently with a spacing of **a,** 2 µm, **c,** 6 µm, and **e,** 10 µm. **b,** Frequency locking between device 1 and device 2 at a 2 µm spacing (I_1 fixed at 2.7 mA). **d,** Frequency locking between device 1 and device 2 at a 6 µm spacing (I_1 fixed at 2.6 mA). **f,** Frequency locking between device 1 and device 2 at

a 10 μm spacing (I_1 fixed at 2.9 mA). As the spacing between the two VO_2 devices becomes larger (from 2 μm to 10 μm), the coupling strength becomes weaker, shown as a smaller frequency locking range (from 22 kHz to 42 kHz at 2 μm; from 21 kHz to 27 kHz at 6 μm; from 25 kHz to 28 kHz at 10 μm).

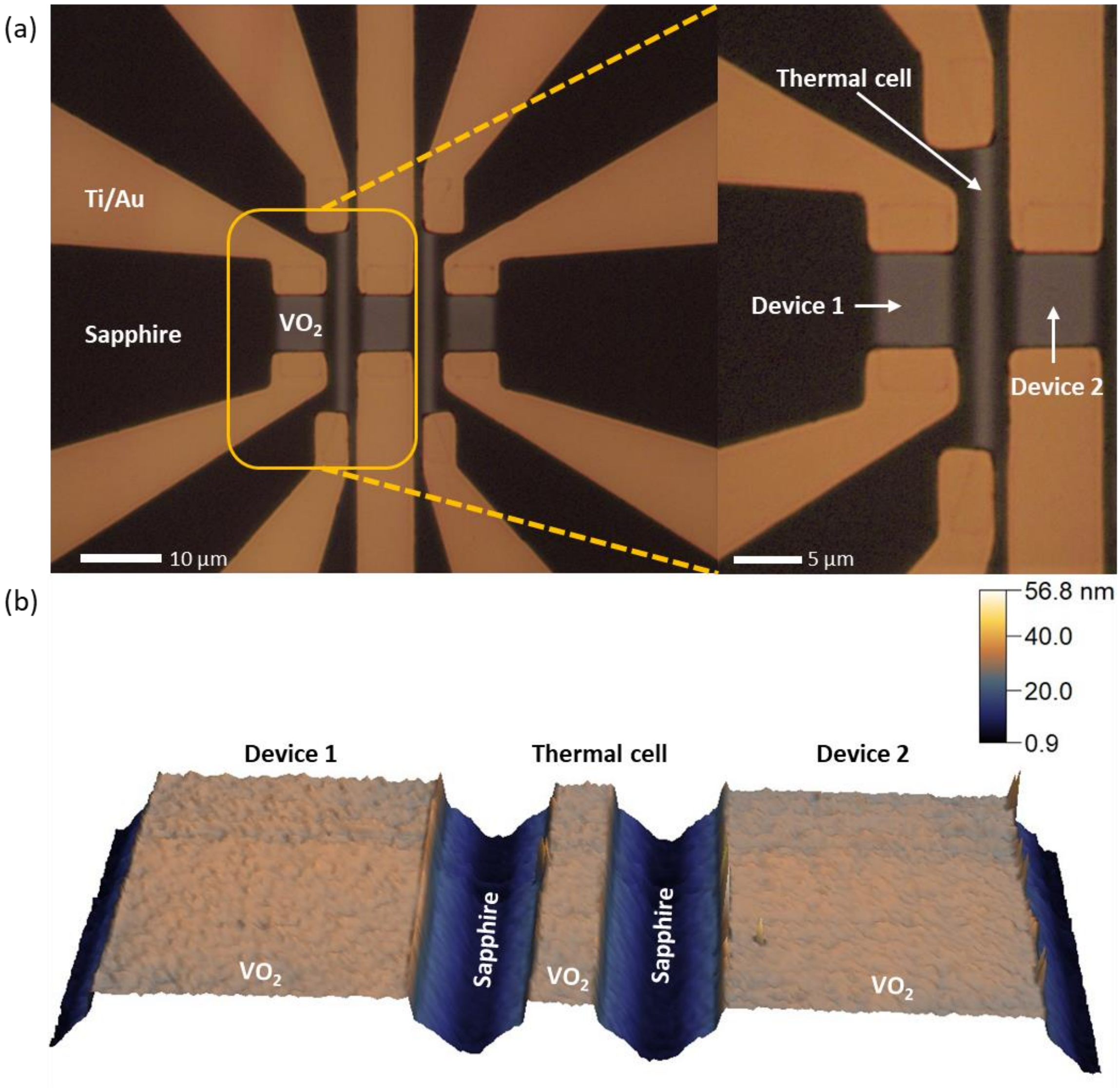

Fig. S9 | Test device set geometry. a, Optical microscopy images of device set for experiments, with overview (left) and zoomed-in view (right). A 1.5 μm wide (21 μm long) VO_2 stripe is placed as a thermal cell for enhancing the thermal coupling between two VO_2 devices with the dimensions of 7 × 6 μm^2. The distance between device 1 and device 2 is 5 μm. **b,** 3D geometry of device 1, device 2 and thermal cell scanned by AFM.

Discussion on enhanced oscillation synchronization via thermal cell

Here, we further discuss how the devices' ambient temperature plays a critical role for the oscillation coupling among neighboring devices including the thermal cell. One common behavior of VO_2 cell is that the oscillation frequency increases by increasing the ambient temperature while reducing the amplitude, as shown from Fig. S10 to Fig. S12. Furthermore, we find that the thermal coupling behavior between devices is mutual that both raise ambient temperature and mutual oscillation frequency, as shown in Fig. S13 and Fig. S14. Applying a constant voltage to the thermal cell has the equivalent effects of raising the ambient temperature that increases the oscillation frequency while lowering the amplitude, as shown in Fig. S15 and Fig. S16. As a result, activating the thermal cell during synchronization helps device 1 (with a constant current I_2 that is lower than the oscillation threshold current) to oscillate at a higher frequency following device 2's frequency as I_2 is gradually increased. As shown in Fig. S17a and b, activating the thermal cell will lead to an increase of the mutual synchronized frequency when device 1 and device 2 are already in a synchronized state ($I_1 = I_2 = 2.5$ mA, $V_{cell} = 0$ V). When device 1 and device 2 are desynchronized ($I_1 = 2.5$ mA, $I_2 = 5$ mA, $V_{cell} = 0$ V), activating the thermal cell will lead to an increase of the oscillation frequency while lowering the oscillation amplitude of device 1, which enables device 1 to be synchronized to device 2, as shown in Fig. S17c and d.

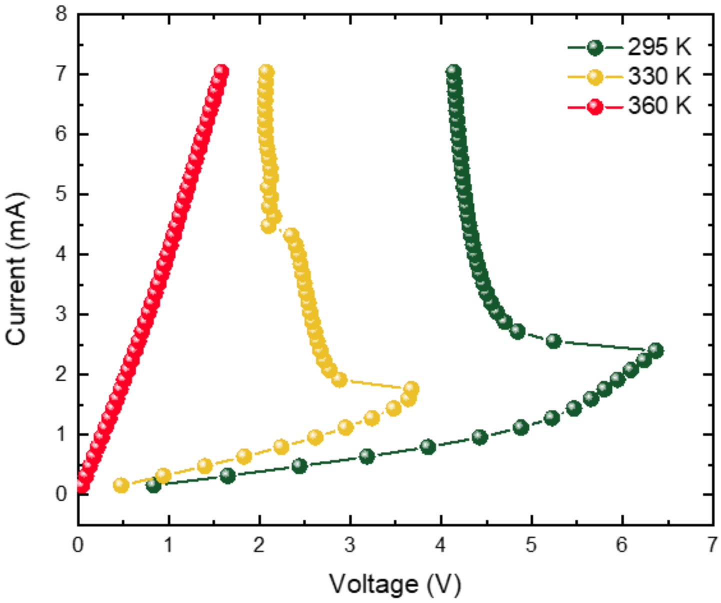

Fig. S10 | *I-V* characteristic of VO_2 device at different temperatures. *I-V* measurements (sweep current and measure voltage, voltage compliance at 20 V) at 295 K, 330 K and 360 K.

330 K is very close to T_c = 340 K: at this temperature a significant decrease of the threshold switching current and voltage from 2.5 mA, 6.4 V (at 295 K) to 1.8 mA, 3.8 V can be observed. At 360 K the material has become fully metallic so that the voltage is proportional to the applied current.

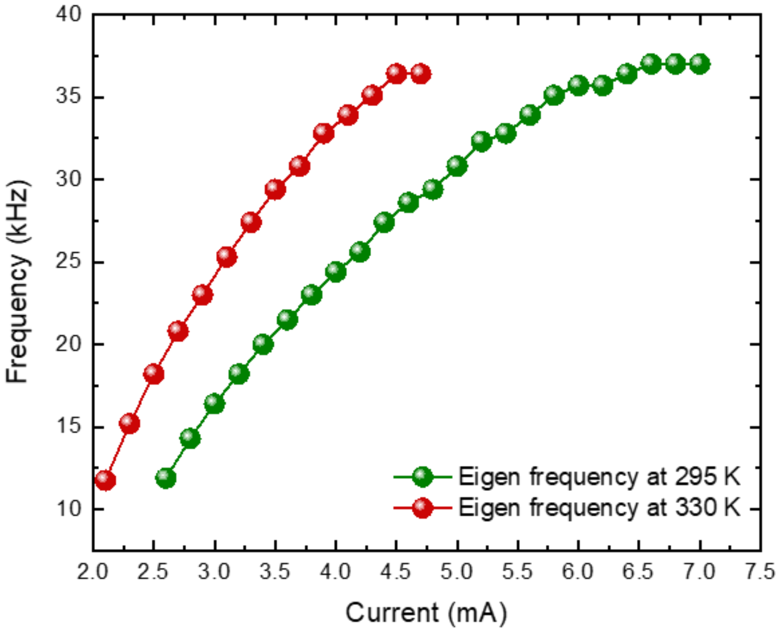

Fig. S11 | Oscillation frequency as a function of applied d.c. current (*I-f*) for VO_2 device (7 × 6 μm^2) at 295 K and 330 K. At higher ambient temperature, with the same supply current the VO_2 device oscillates at a higher frequency but the tunable frequency range becomes narrower. Additionally, the threshold current value where VO_2 device starts to oscillate decreases at higher temperatures.

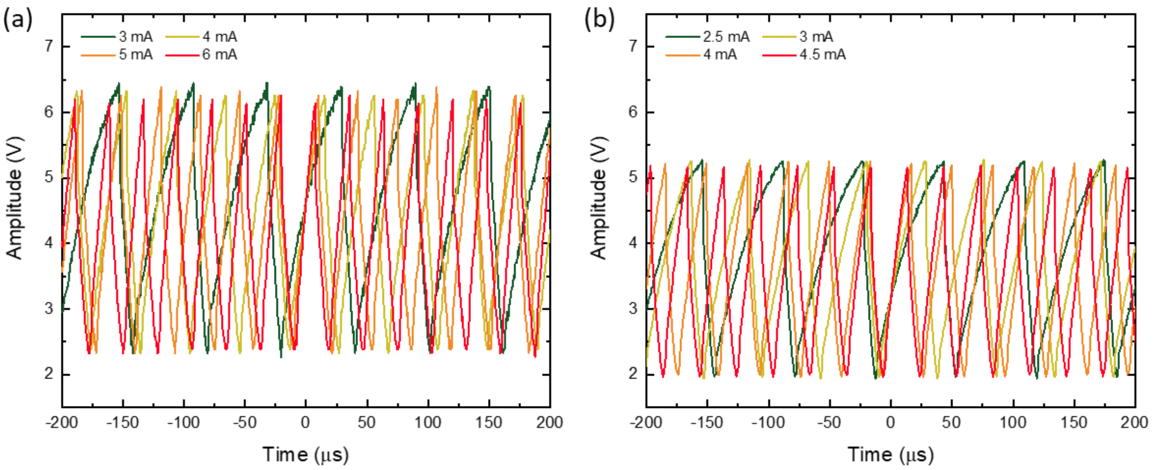

Fig. S12 | Single device oscillation waveform for different supply currents and temperatures. a, Single device (7 × 6 μm^2) oscillation waveform at 3 mA, 4 mA, 5 mA, 6 mA at 295 K. **b,** Single device (7 × 6 μm^2) oscillation waveform at 2.5 mA, 3 mA, 4 mA, 4.5 mA at 330 K. At the same temperature with different supply currents the oscillation amplitude hardly changes. At higher temperatures the amplitude becomes smaller (at 295 K: peak-to-peak value is about 4.1 V, while at 330 K: peak-to-peak value is about 3.2 V).

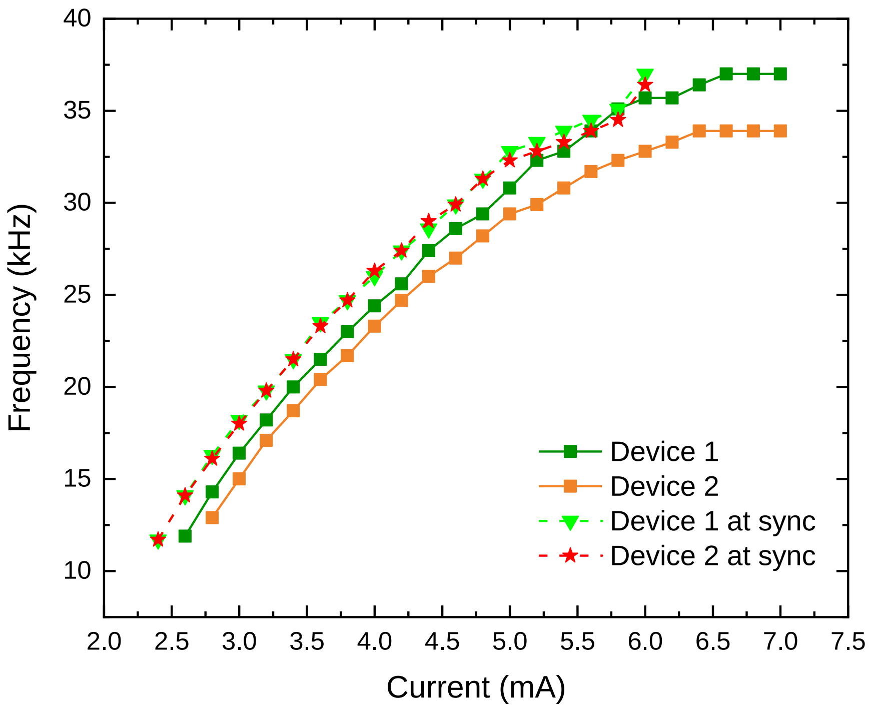

Fig. S13 | Oscillation frequency as a function of applied d.c. current (*I*-*f*) of device 1 (7 × 6 μm^2) and device 2 (7 × 6 μm^2) when they are oscillating independently, and when they are synchronized ($I_1 = I_2$). At synchronization, both device 1 and device 2 oscillate at a higher

frequency but the tunable frequency range becomes narrower. Additionally, the threshold current value where device 1 and device 2 start to oscillate decreases during synchronization, indicating a higher ambient temperature during synchronization than when they are oscillating independently.

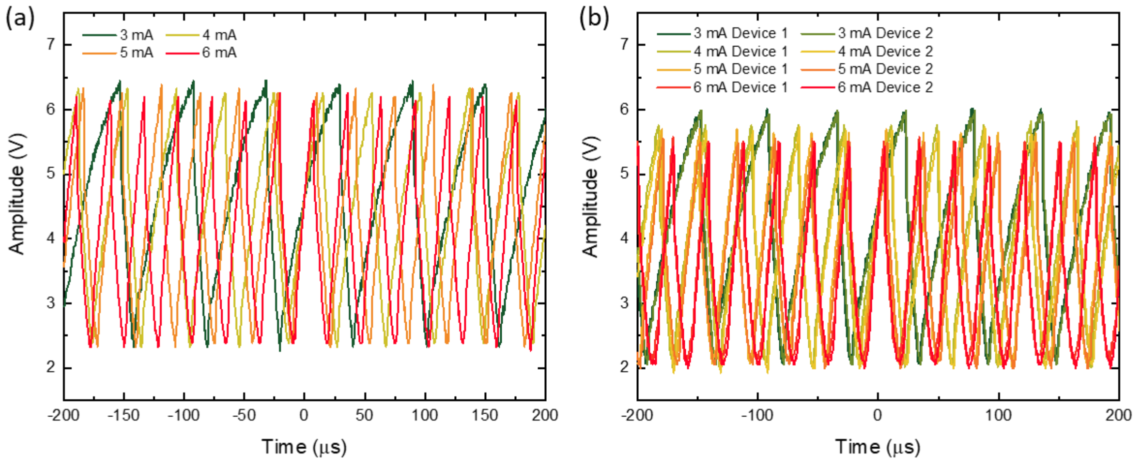

Fig. S14 | Single device oscillation waveform at different supply currents and double device synchronized oscillation waveforms for different supply currents. a, Single device (7 × 6 μm^2) oscillation waveform at 3 mA, 4 mA, 5 mA, 6 mA at 295 K. At same temperature with different supply currents the oscillation amplitude hardly changes (peak-to-peak value is about 4.1 V). **b,** Device 1 (7 × 6 μm^2) and device 2 (7 × 6 μm^2) synchronized oscillation waveform at 3 mA, 4 mA, 5 mA, 6 mA at 295 K. At synchronization the amplitude becomes smaller (at 3 mA: peak-to-peak value is about 3.9 V, at 4 mA: peak-to-peak value is about 3.7 V, at 5 mA: peak-to-peak value is about 3.6 V, at 6 mA: peak-to-peak value is about 3.5 V).

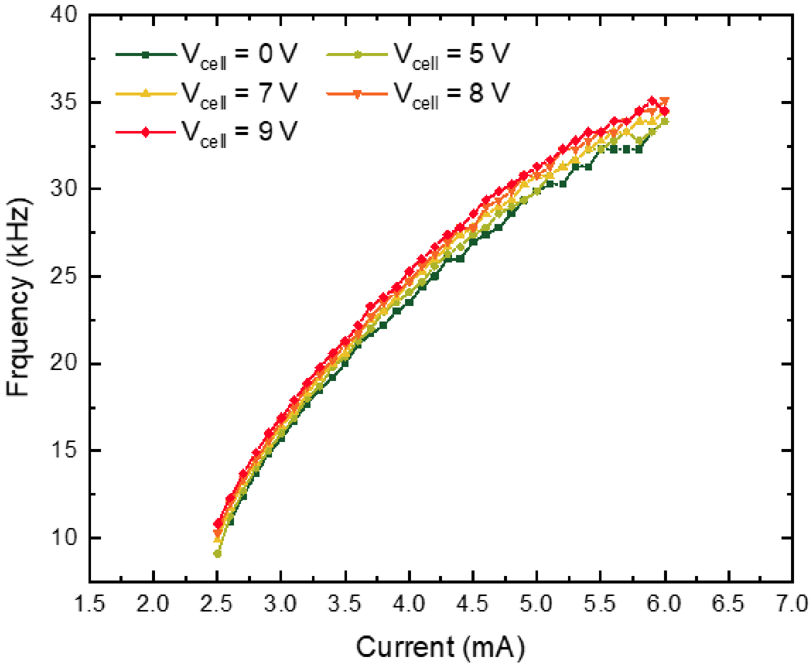

Fig. S15 | Oscillation frequency as a function of applied d.c. current (*I*-*f*) for VO_2 device ($7 \times 6\ \mu m^2$) at 295 K with different thermal cell voltages V_{cell}. It can be observed that with higher thermal cell voltage, the device oscillates at a higher frequency.

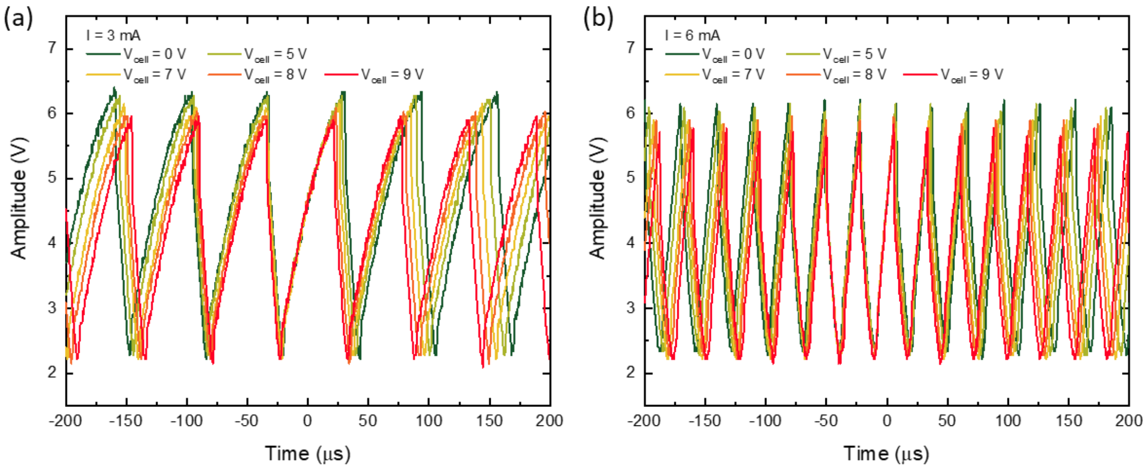

Fig. S16 | Single device oscillation waveform at different supply currents and thermal cell voltages. a, Single device ($7 \times 6\ \mu m^2$) oscillation waveform at 3 mA at 295 K for different thermal cell voltages V_{cell}. **b,** Single device ($7 \times 6\ \mu m^2$) oscillation waveform at 6 mA at 295 K for different thermal cell voltage V_{cell}. For higher thermal cell voltages, the device oscillates at a higher frequency with a smaller amplitude.

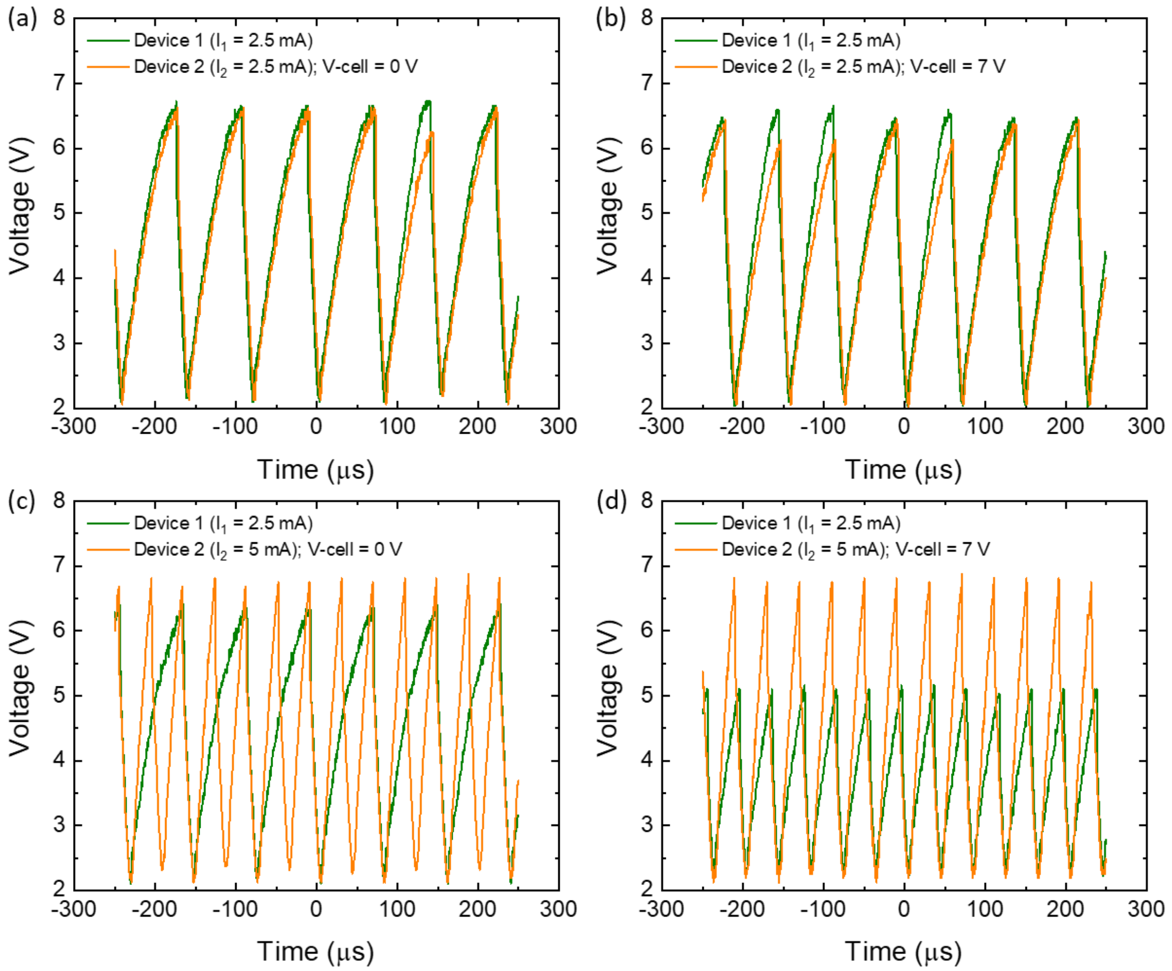

Fig. S17 | Double device oscillation waveforms under synchronization and desynchronization situations. a, Device 1 (7 × 6 μm^2) and device 2 (7 × 6 μm^2) synchronized oscillation waveforms at $I_1 = I_2 = 2.5$ mA, $V_{cell} = 0$ V. **b,** Device 1 and device 2 synchronized oscillation waveforms at $I_1 = I_2 = 2.5$ mA, $V_{cell} = 7$ V. **c,** Device 1 and device 2 desynchronized oscillation waveforms at $I_1 = 2.5$ mA, $I_2 = 5$ mA, $V_{cell} = 0$ V. **d,** Device 1 and device 2 synchronized oscillation waveforms at $I_1 = 2.5$ mA, $I_2 = 5$ mA, $V_{cell} = 7$ V.

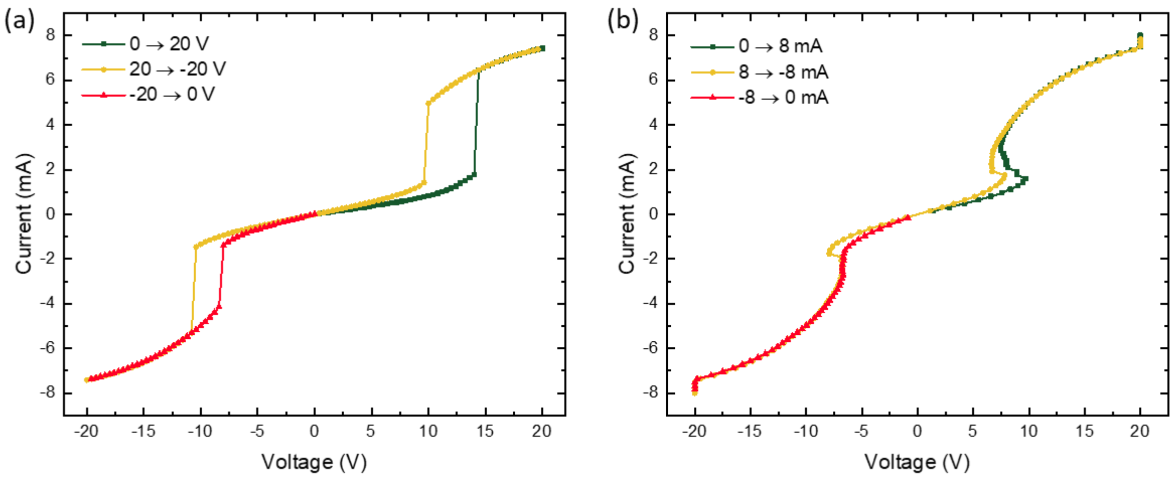

Fig. S18 | Voltage-mode (*V-I*) and current-mode (*I-V*) measurements for a thermal cell. a, Thermal cell *V-I* (sweep voltage and measure current, current compliance at 10 mA) and **b,** *I-V* (sweep current and measure voltage, voltage compliance at 20 V) curves.

Power consumption of microscopic VO_2 oscillators with thermal cell

The device set includes a 1.5 μm wide (21 μm long) VO_2 stripe as a thermal cell between two VO_2 oscillators, each with dimensions of 7 × 6 μm^2. The distance between device 1 and device 2 is 5 μm. Gray line shows the total power (P_{sum}) when they are oscillating independently: P_{sum} = Power from device 1 (P_1) (I_1 = 2.4 mA, I_2 = 0) + Power from device 2 (P_2) (I_2 = 4, 5, 5.5 mA, I_1 = 0). Yellow line shows P_{sum} when they are oscillating with the coupling effect at V_{cell} = 0 V: P_{sum} = P_1 (I_1 = 2.4 mA, I_2 = 4, 5, 5.5 mA) + P_2 (I_2 = 4, 5, 5.5 mA, I_1 = 2.4 mA). Green line shows P_{sum} when they are oscillating with the coupling effect at V_{cell} = 5 V: P_{sum} = P_1 (I_1 = 2.4 mA, I_2 = 4, 5, 5.5 mA) + P_2 (I_2 = 4, 5, 5.5 mA, I_1 = 2.4 mA) + Power from thermal cell (P_{cell}) (V_{cell} = 5 V). Orange line shows the total power when they are oscillating with the coupling effect at V_{cell} = 7 V: P_{sum} = P_1 (I_1 = 2.4 mA, I_2 = 4, 5, 5.5 mA) + P_2 (I_2 = 4, 5, 5.5 mA, I_1 = 2.4 mA) + P_{cell} (V_{cell} = 7 V). Light red line shows the total power when they are oscillating with the coupling effect at V_{cell} = 7.5 V: P_{sum} = P_1 (I_1 = 2.4 mA, I_2 = 4, 5, 5.5 mA) + P_2 (I_2 = 4, 5, 5.5 mA, I_1 = 2.4 mA) + P_{cell} (V_{cell} = 7.5 V). Dark red line shows the total power when they are oscillating with the coupling effect at V_{cell} = 8 V: P_{sum} = P_1 (I_1 = 2.4 mA, I_2 = 4, 5, 5.5 mA) + P_2 (I_2 = 4, 5, 5.5 mA, I_1 = 2.4 mA) + P_{cell} (V_{cell} = 8 V). The power of a single oscillator is calculated as follows: The oscillating voltage waveform under a certain current value is collected. Then the voltage value is averaged over time (500 μs) then multiplied by the current

value to obtain the average power consumption. The power of the thermal cell is calculated approximately from the measurements in Fig. S18.

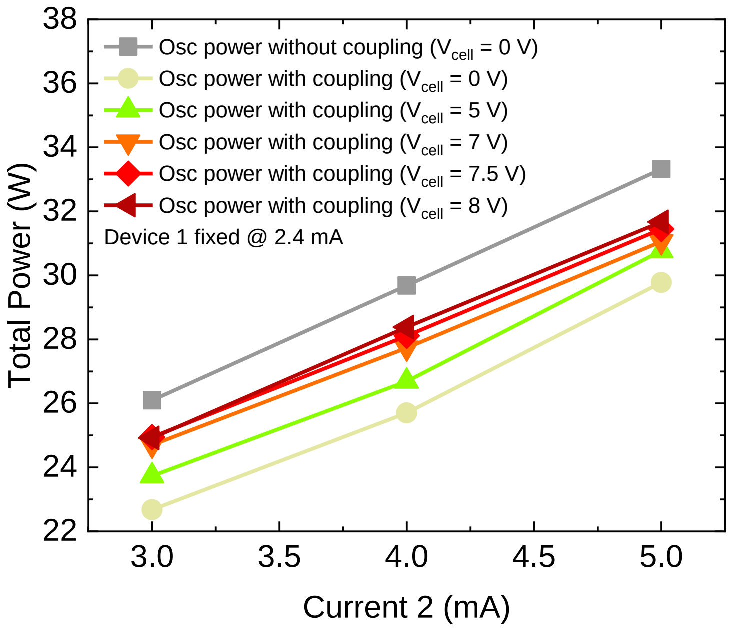

Fig. S19 | Comparison of power consumption from device 1 and device 2 for different V_{cell}.

Nanoscopic VO_2 oscillators with tunable synchronization range

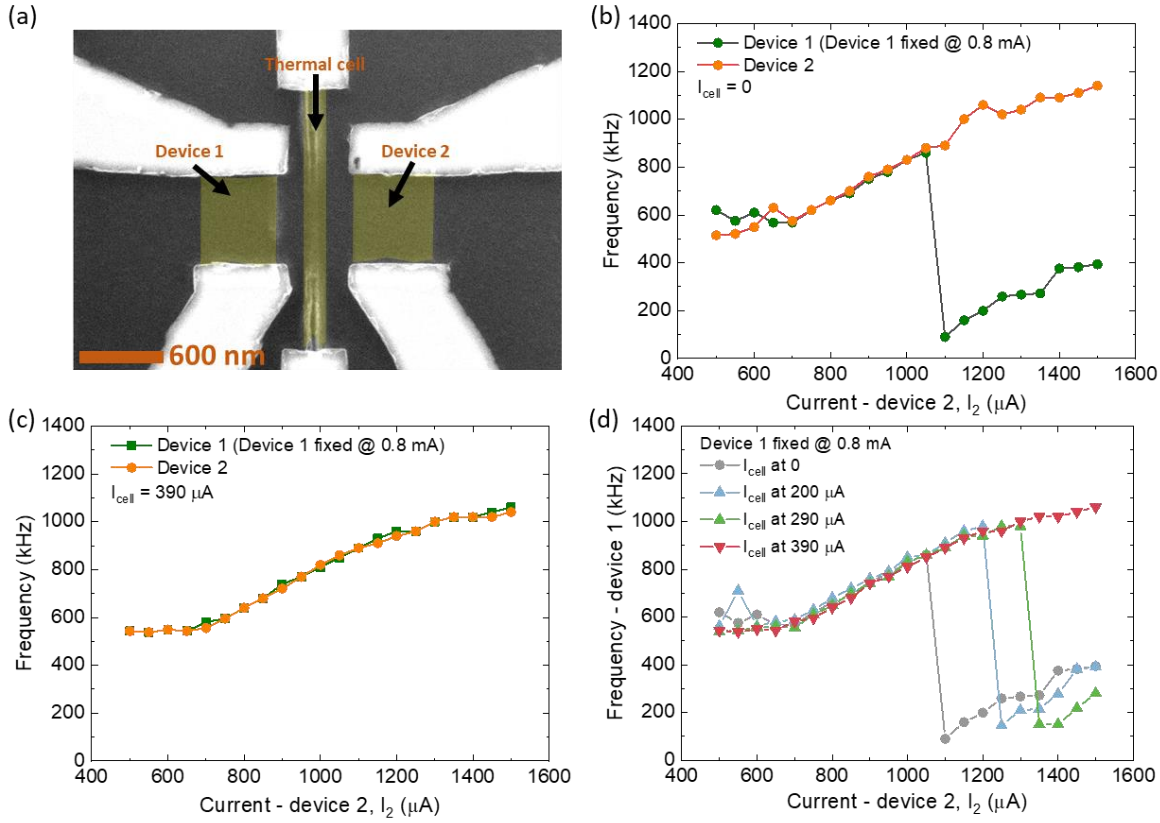

Fig. S20 | VO_2 nano oscillators with tunable thermal triggering. a, SEM image of a fabricated nanoscopic device. The device set includes a 120 nm wide (2 μm long) VO_2 nano wire as a thermal cell, and two VO_2 oscillators with dimensions of 700 × 600 nm^2. The distance between device 1 and device 2 is 600 nm. The distance between thermal cell and device 1 (or device 2) is 240 nm. Yellow shading indicates VO_2 cells (Au contacts in white). **b**, Frequency locking behavior at I_{cell} = 0 μA. For this measurement, the current to device 1 is fixed at 0.8 mA while the current to device 2 is increased. In this case, the frequency locking between device 1 and 2 holds from 700 kHz to 1.05 MHz and then breaks down. **c**, Frequency locking behavior at I_{cell} = 390 μA. In this case, the frequency locking breaks down above 1.5 MHz. **d**, Comparison of synchronization frequency of device 1 (fixed at 0.8 mA) as I_{cell} is varied (from 0 to 390 μA).

Discussion on generating different oscillation states from coupled VO_2 devices

The key to generating different oscillation states is to use the supply current and thermal cell to bias the starting point of the oscillation cycle in the R-T hysteresis loop. The oscillation state in Fig. S21a: Device 1 is supplied with constant current of 2.5 mA while the thermal cell is deactivated. The ambient temperature isn't significantly raised so device 1 oscillates at a low frequency with large amplitude; The oscillation state in Fig. S21b: Device 1 is supplied with constant large current of 6.2 mA with the activated thermal cell. Meanwhile, device 2 is supplied with large current (I_2 = 5 mA) and oscillates at a high frequency. Due to the significant rise of ambient temperature by both synchronization and the thermal cell, the high resistance state of device 1 has been biased closely to the metallic point (in the R-T hysteresis loop) and this results in a smaller peak-to-peak amplitude (< 1 V) and high frequency; The same also applies to the oscillation state in Fig. S21c when device 1 is supplied with a constant current of 6.4 mA while the thermal cell is activated, and device 2 oscillates at a low frequency (I_2 = 2.4 mA); The oscillation state in Fig. S21d: Device 1 is supplied with a constant current of 6.4 mA while device 2 is supplied with a large current (I_2 = 5.2 mA) and oscillates at high frequency. The activation of the thermal cell will lead to overheating of both devices so they reach the metallic state. As a result, there will be no more oscillations, as shown in Fig. S21d.

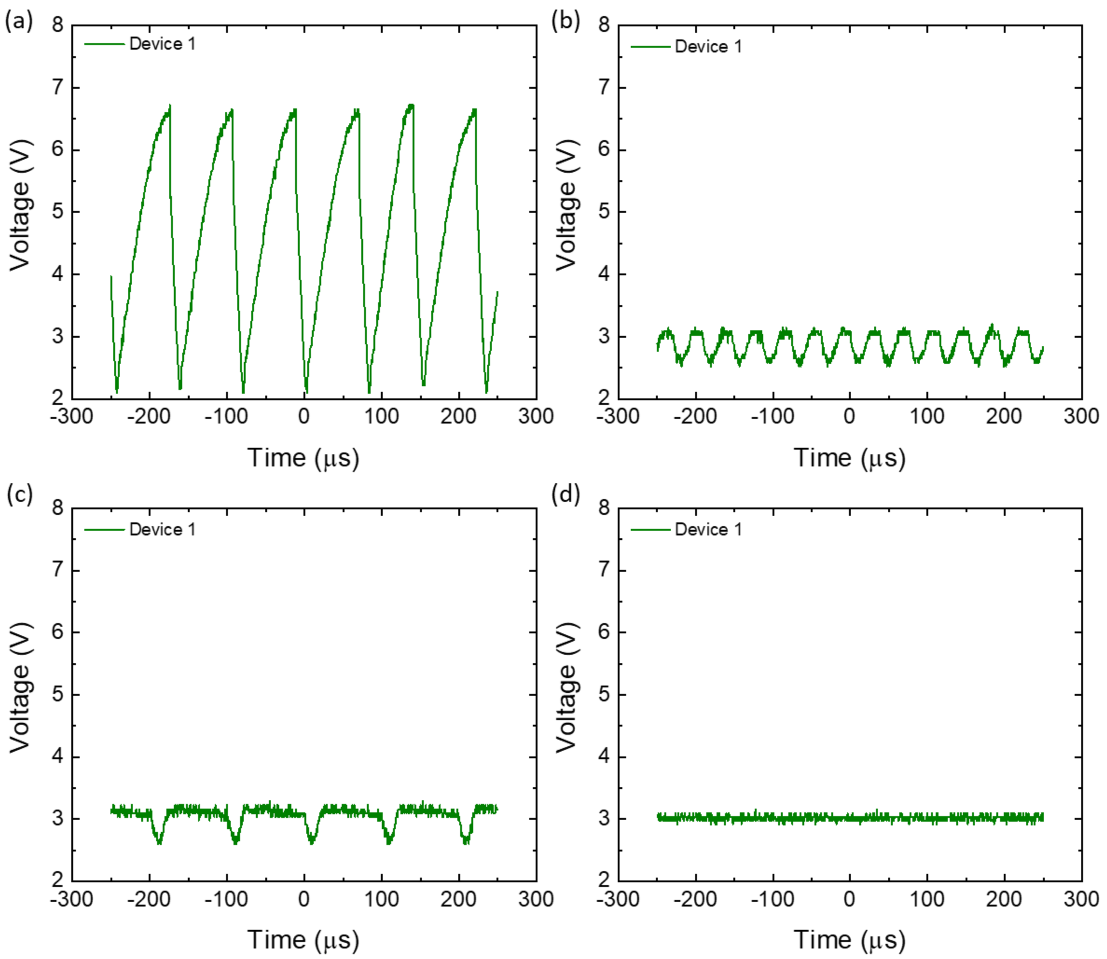

Fig. S21 | Logic "0" represented by different oscillation states of VO_2 device 2. a, Oscillation state with low frequency and large amplitude. **b,** Oscillation state with high frequency and small amplitude. **c,** Oscillation state with low frequency and small amplitude. **d,** No oscillation (fully metallic state).

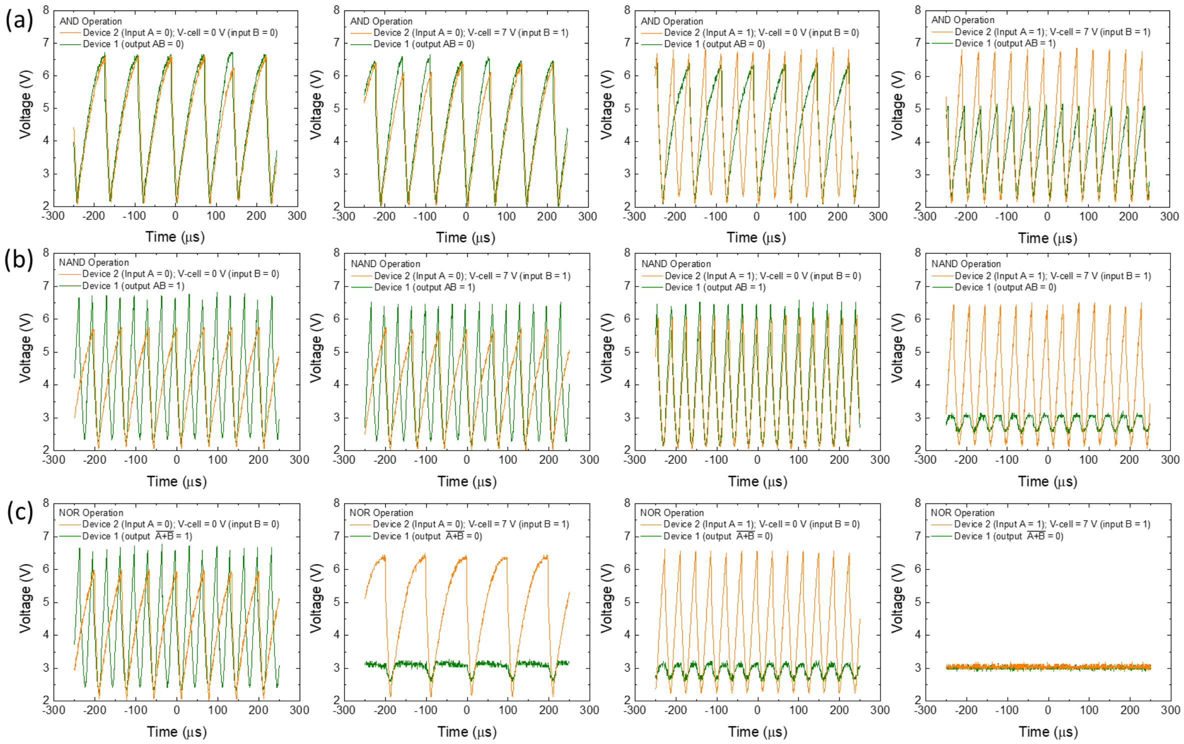

Fig. S22 | Output waveforms of Boolean logic gates. a, AND gate operation. **b**, NAND gate operation. **c**, NOR gate operation.

(a) AND operation:

Input A (Device 2)	Input B (V_{cell})	Output AB (Device 1 fixed at 2.5 mA)
**0** (f = 11.9 kHz, $V_{pk\text{-}pk}$ = 4.4 V at 2.5 mA)	**0** (V_{cell} = 0 V)	**0** (f = 11.9 kHz, $V_{pk\text{-}pk}$ = 4.4 V)
**0** (f = 11.9 kHz, $V_{pk\text{-}pk}$ = 4.4 V at 2.5 mA)	**1** (V_{cell} = 7 V)	**0** (f = 14.3 kHz, $V_{pk\text{-}pk}$ = 4.4 V)
**1** (f = 30.8 kHz, $V_{pk\text{-}pk}$ = 4.5 V at 5 mA)	**0** (V_{cell} = 0 V)	**0** (f = 12.7 kHz, $V_{pk\text{-}pk}$ = 4.4 V)
**1** (f = 30.8 kHz, $V_{pk\text{-}pk}$ = 4.5 V at 5 mA)	**1** (V_{cell} = 7 V)	**1** (f = 24.4 kHz, $V_{pk\text{-}pk}$ = 2.7 V)

(b) NAND operation:

Input A (Device 2)	Input B (V_{cell})	Output $\overline{AB}$ (Device 1 fixed at 6.2 mA)
**0** (f = 11 kHz, $V_{pk\text{-}pk}$ = 4.4 V at 2.4 mA)	**0** (V_{cell} = 0 V)	**1** (f = 30.3 kHz, $V_{pk\text{-}pk}$ = 4.4 V)
**0** (f = 11 kHz, $V_{pk\text{-}pk}$ = 4.4 V at 2.4 mA)	**1** (V_{cell} = 7 V)	**1** (f = 29.4 kHz, $V_{pk\text{-}pk}$ = 4.1 V)
**1** (f = 30.8 kHz, $V_{pk\text{-}pk}$ = 4.5 V at 5 mA)	**0** (V_{cell} = 0 V)	**1** (f = 30.3 kHz, $V_{pk\text{-}pk}$ = 4.3 V)
**1** (f = 30.8 kHz, $V_{pk\text{-}pk}$ = 4.5 V at 5 mA)	**1** (V_{cell} = 7 V)	**0** (f = 24.4 kHz, $V_{pk\text{-}pk}$ = 0.6 V)

(c) NOR operation:

Input A (Device 2)	Input B (V_{cell})	Output $\overline{A+B}$ (Device 1 fixed at 6.4 mA)
**0** (f = 11.9 kHz, $V_{pk\text{-}pk}$ = 4.4 V at 2.5 mA)	**0** (V_{cell} = 0 V)	**1** (f = 31.3 kHz, $V_{pk\text{-}pk}$ = 4.2 V)
**0** (f = 11.9 kHz, $V_{pk\text{-}pk}$ = 4.4 V at 2.5 mA)	**1** (V_{cell} = 7 V)	**0** (f = 10.1 kHz, $V_{pk\text{-}pk}$ = 0.6 V)
**1** (f = 32.3 kHz, $V_{pk\text{-}pk}$ = 4.4 V at 5.2 mA)	**0** (V_{cell} = 0 V)	**0** (f = 27 kHz, $V_{pk\text{-}pk}$ = 0.4 V)
**1** (f = 32.3 kHz, $V_{pk\text{-}pk}$ = 4.4 V at 5.2 mA)	**1** (V_{cell} = 7 V)	**0** (no oscillation)

Supplementary data table. T2 | Calculation table of Boolean logic gates. Output logic “0” and “1” are represented by different oscillation states of VO_2 device 1. **a,** AND gate. **b,** NAND gate. **c,** NOR gate.

Cascade synchronization among VO_2 oscillators and the thermal cell

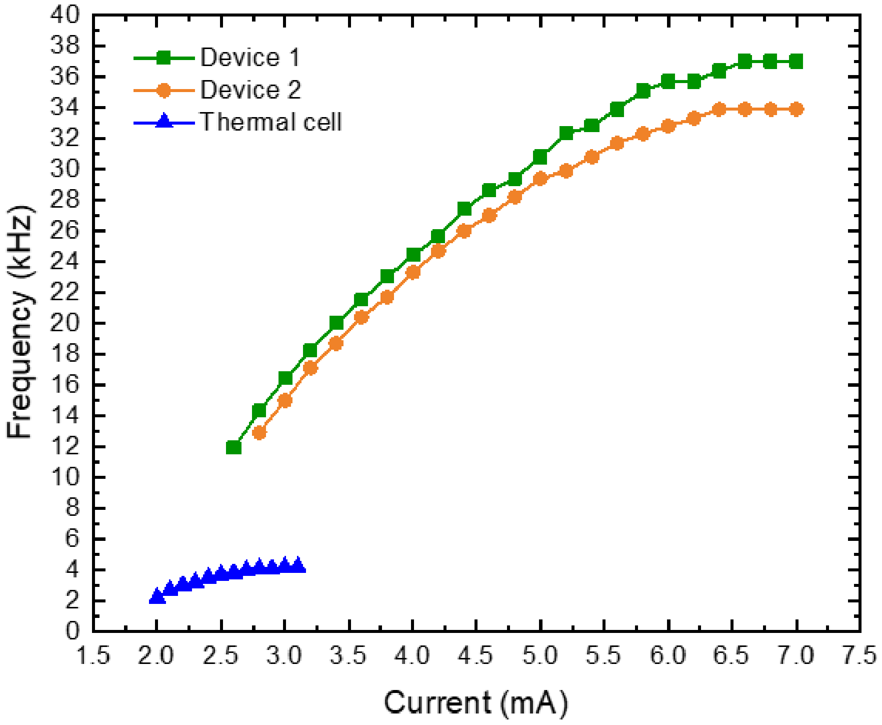

Fig. S23 | Oscillation frequency as a function of applied d.c. current (*I*-*f*). Device 1 (VO_2 cell 2, 7 × 6 μm^2) shown as green line, device 2 (VO_2 cell 1, 7 × 6 μm^2) shown as orange line and thermal cell (1.5 × 21 μm^2) shown as blue line.

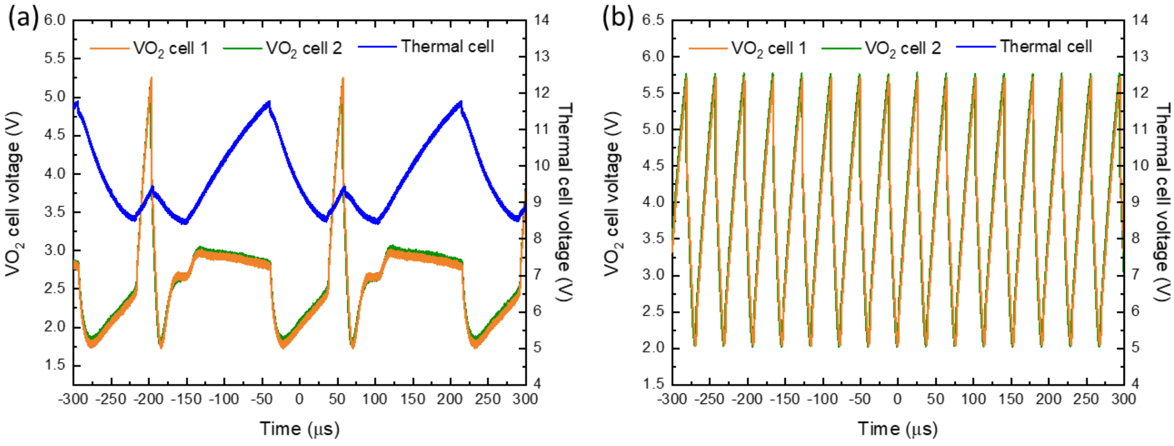

Fig. S24 | VO_2 neuron firing mode 1. a, Spiking neuron firing mode 1 at I_1 = 4 mA (applied current of VO_2 cell 1), I_2 = 4 mA (applied current of VO_2 cell 2) and I_{cell} = 2.3 mA (applied current of thermal cell). **b**, I_1 = 4 mA, I_2 = 4 mA and I_{cell} = 0 mA.

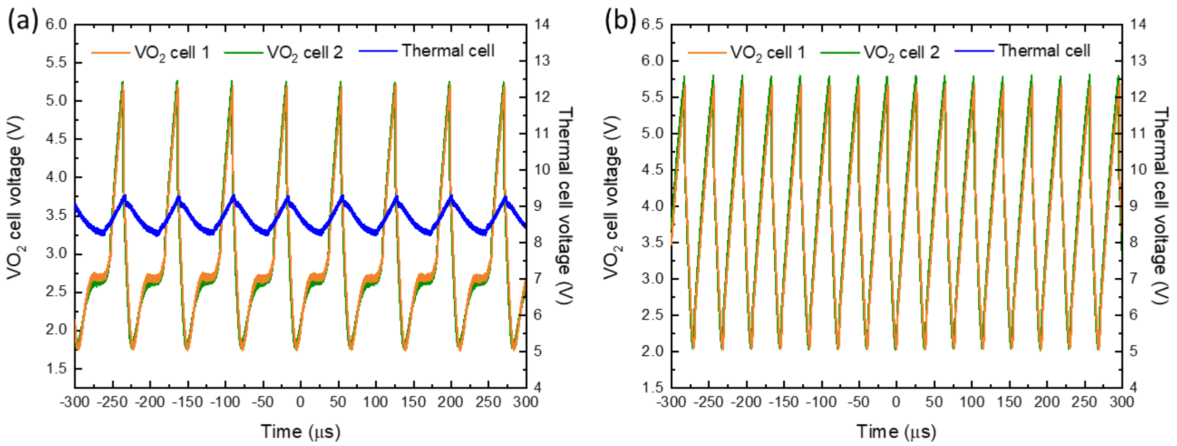

Fig. S25 | VO$_2$ neuron firing mode 2. a, Spiking neuron firing mode 2 at I_1 = 3.9 mA (applied current of VO$_2$ cell 1), I_2 = 4 mA (applied current of VO$_2$ cell 2) and I_{cell} = 2.3 mA (applied current of thermal cell). **b**, I_1 = 3.9 mA, I_2 = 4 mA and I_{cell} = 0 mA.

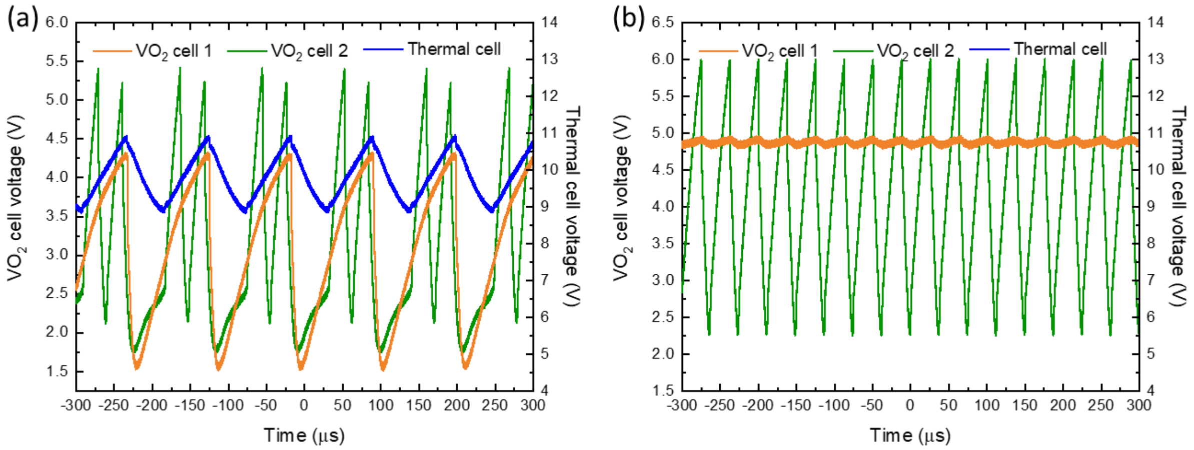

Fig. S26 | VO$_2$ neuron firing mode 3. a, Spiking neuron firing mode 3 at I_1 = 1.7 mA (applied current of VO$_2$ cell 1), I_2 = 4 mA (applied current of VO$_2$ cell 2) and I_{cell} = 2.3 mA (applied current of thermal cell). **b**, I_1 = 1.7 mA, I_2 = 4 mA and I_{cell} = 0 mA.

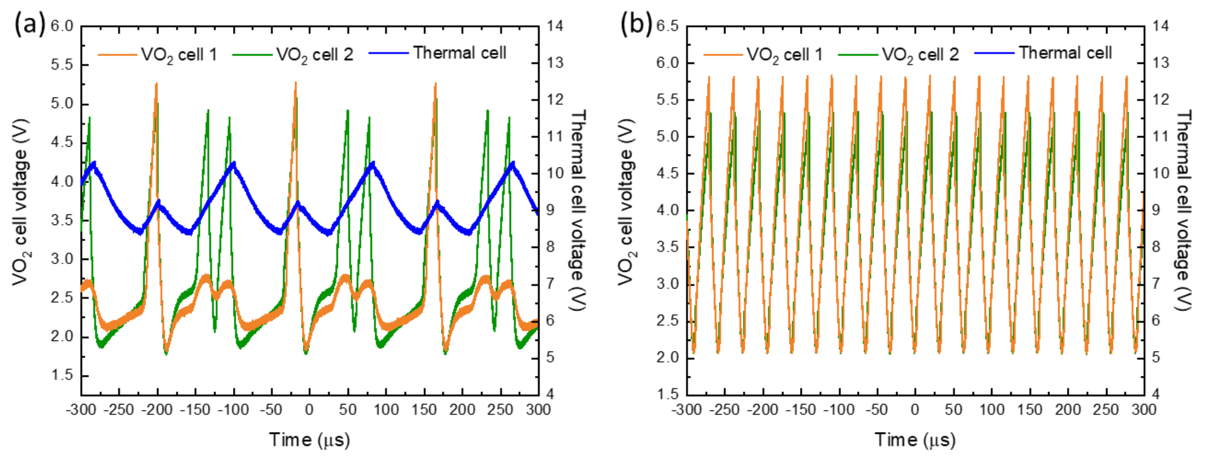

Fig. S27 | VO_2 neuron firing mode 4. a, Spiking neuron firing mode 4 at I_1 = 5.2 mA (applied current of VO_2 cell 1), I_2 = 4 mA (applied current of VO_2 cell 2) and I_{cell} = 2.3 mA (applied current of thermal cell). **b**, I_1 = 5.2 mA, I_2 = 4 mA and I_{cell} = 0 mA.

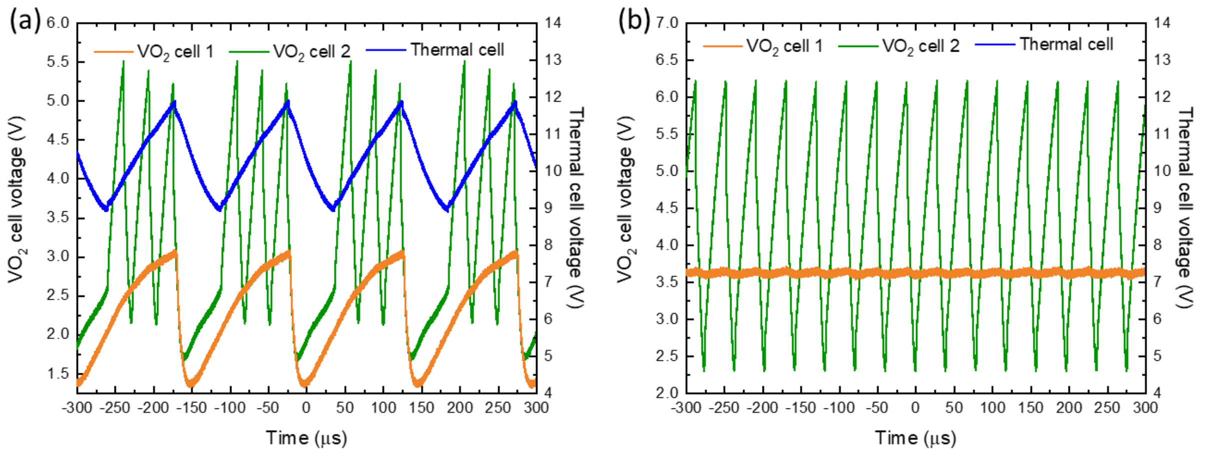

Fig. S28 | VO_2 neuron firing mode 5. a, Spiking neuron firing mode 5 at I_1 = 1 mA (applied current of VO_2 cell 1), I_2 = 4 mA (applied current of VO_2 cell 2) and I_{cell} = 2.3 mA (applied current of thermal cell). **b**, I_1 = 1 mA, I_2 = 4 mA and I_{cell} = 0 mA.

References

[R1] Dickson, R.W. and Schreiber, E. *J. Research of NBS.* **v77A**, No. 4, p391 (1973).

[R2] Archer, D.A. *J. Phys. Chem. Ref. Data*. **v22**, No. 6, p1441 (1993).

[R3] Burghartz, St. and Schulz, B. *J. Nucl. Mater.*, v212-215, p1065 (1994)

[R4] Taylor, D. *Br. ceram., Trans. j.* **v83**, No. 2, p32 (1984).

[R5] Kizuka, H., el al. *Jpn. J. Appl. Phys.* **54**, 053201 (2015).
